# Intra-Cyclic Variation of Velocity in Swimming: Association and Consistency Between the Most-Used Measurements

**DOI:** 10.3390/jfmk11030328

**Published:** 2026-08-23

**Authors:** Jorge E. Morais, Tiago M. Barbosa

**Affiliations:** 1 Department of Sport Sciences, Universidade Politécnica de Bragança, 5300-253 Bragança, Portugal; barbosa@unipb.pt; 2 Research Centre for Active Living and Wellbeing (LiveWell), Universidade Politécnica de Bragança, 5300-253 Bragança, Portugal

**Keywords:** swimming, biomechanics, velocity variation, butterfly, association, consistency

## Abstract

**Background and Objectives**: Intra-cyclic variation in swimming velocity is one of the variables most commonly used to assess swimming performance, providing simple, straightforward information for coaches and swimmers. Therefore, the main aim of this study was to assess the consistency and association between the two most commonly used methods for calculating the intra-cyclic variation in velocity in swimming (coefficient of variation—dv1; and range: the difference between the maximum and minimum instantaneous velocities—dv2). **Methods**: Twenty-one young butterfly swimmers (11 males and 10 females) with an average age of 17.3 ± 2.8 years were recruited for analysis. The measurements were done in the butterfly stroke at maximal velocities. The consistency and association criteria included the intraclass correlation coefficient (ICC) and simple linear regression between the two methods. **Results**: The ICC between dvs was excellent for the overall sample (ICC = 0.972, *p* < 0.001), males (ICC = 0.978, *p* < 0.001), and females (ICC = 0.955, *p* < 0.001). The linear regression between methods was very high (overall: R^2^ = 0.93, *p* < 0.001; males: R^2^ = 0.91, *p* < 0.001; females: R^2^ = 0.86, *p* < 0.001). **Conclusions**: Overall, coaches and researchers should be aware that although both methods yielded highly concordant results, they are based on different mathematical formulations and quantify distinct characteristics of the velocity signal. Therefore, their consistency and association should not be interpreted as mathematical equivalence or universal interchangeability.

## 1. Introduction

The analysis of intra-cyclic variation in swimming velocity (IVV), also known as speed fluctuation (dv—used from now on as an acronym for velocity fluctuations), has emerged as a pertinent parameter in swimming biomechanics [1,2]. Even nowadays, it is one of the most reported parameters in swimming biomechanics [3]. This represents the fluctuations in a swimmer’s instantaneous velocity throughout the stroke cycle. The balance between propulsion and drag determines the acceleration, thereby the mean swimming velocity and the amplitude of its intra-cyclic fluctuations [4]. The dv is historically associated with efficiency measurements [5,6], whereas, as intensity increases, efficiency is expected to decrease, as observed in human locomotion [7]. However, several studies have used it to examine its association at maximal intensities, noting that smaller dvs were associated with better performance [8,9]. In the butterfly stroke, greater velocity variation is associated with greater energy expenditure and reduced efficiency, whereas a more stable velocity is associated with a smoother, more economical stroke pattern [10]. These findings were also noted for the other three swim strokes (backstroke, breaststroke, and front crawl) [11]. Thus, dv provides critical insights into the efficiency and effectiveness of stroke mechanics.

From a methodological standpoint, several techniques have been developed and used to collect data for the dv calculation: (i) video analysis; (ii) mechanical devices; and (iii) wearables, like inertial measurement units [3]. All these methods measure and acquire the instantaneous velocity for further analysis. After having the velocity–time series, several methods are reported to calculate the dv: (i) the coefficient of variation (CV); (ii) the difference between the maximum and minimum instantaneous velocity (range); (iii) the ratio of mean velocity over the difference between the maximum and minimum instantaneous velocity, and; (iv) the ratio of the minimum and maximum velocities over intra-cyclic mean velocity [3]. In a review study about the dv, Fernandes and co-workers reported that most studies used the CV to interpret the dv, followed by the difference between the maximum and minimum instantaneous velocity (range) [3].

Nonetheless, it was argued that the dv calculated as being the CV could give misleading outputs [12]. The authors stated that results should always be interpreted alongside the mean and standard deviation to avoid misleading conclusions and feedback. Arguably, CV is likely biased by mean velocity [12]. Regarding the difference between the maximum and minimum instantaneous velocity (range), others have used this approach to quantify velocity fluctuations [13,14]. However, the range provides only a narrow summary of variability because it is based solely on two observations and does not account for the distribution of the remaining data. Consequently, it is highly influenced by extreme values and offers a limited representation of overall dispersion. Methodological texts also highlight that identical ranges can occur across datasets with substantially different internal variability, underscoring the conceptual limitations of the range relative to measures such as the CV [15].

Given the role that variations play in swimming, it seems of major importance to understand how they can be interpreted using these two main methods (i.e., as the CV and the difference between the maximum and minimum instantaneous velocity range). This will provide researchers and coaches with deeper insights into the main differences between them and whether these can be used concurrently to further interpret them. Therefore, the main aim of this study was to assess the association and consistency between these two methods for characterizing dv. It was hypothesized that CV and range would be positively associated, but would not provide consistent relative information across swimmers, as they represent different dimensions of dv, i.e., CV reflects overall relative variability, whereas range reflects the amplitude between extreme velocity values.

## 2. Materials and Methods

### 2.1. Participants

For this purpose, the sample was composed of 21 young butterfly swimmers (11 males: 18.8 ± 2.9 years, 76.6 ± 9.3 kg of body mass, 183.8 ± 8.6 cm of height, 186.5 ± 7.4 cm of arm span, 584.0 ± 103.1 World Aquatic Points in the 50 m butterfly event; 10 females: 15.6 ± 1.4 years, 60.6 ± 6.3 kg of body mass, 169.2 ± 7.1 cm of height, 171.5 ± 6.7 cm of arm span, 505.9 ± 89.6 World Aquatic Points in the 50 m butterfly event). These swimmers regularly competed in regional and national events and were supervised by the national team staff, including Tier 2 and 3 athletes [16]. As inclusion criteria, participants were required to be butterfly specialists, free from injury, and to have trained regularly during the previous six months. Coaches and/or parents and the swimmers gave their consent/assent to participate in this study. All human research procedures were conducted in accordance with the Declaration of Helsinki. The Polytechnic Ethics Committee approved the study design (P547664-R678573-D2082555).

### 2.2. Experimental Design

**Figure 1:**
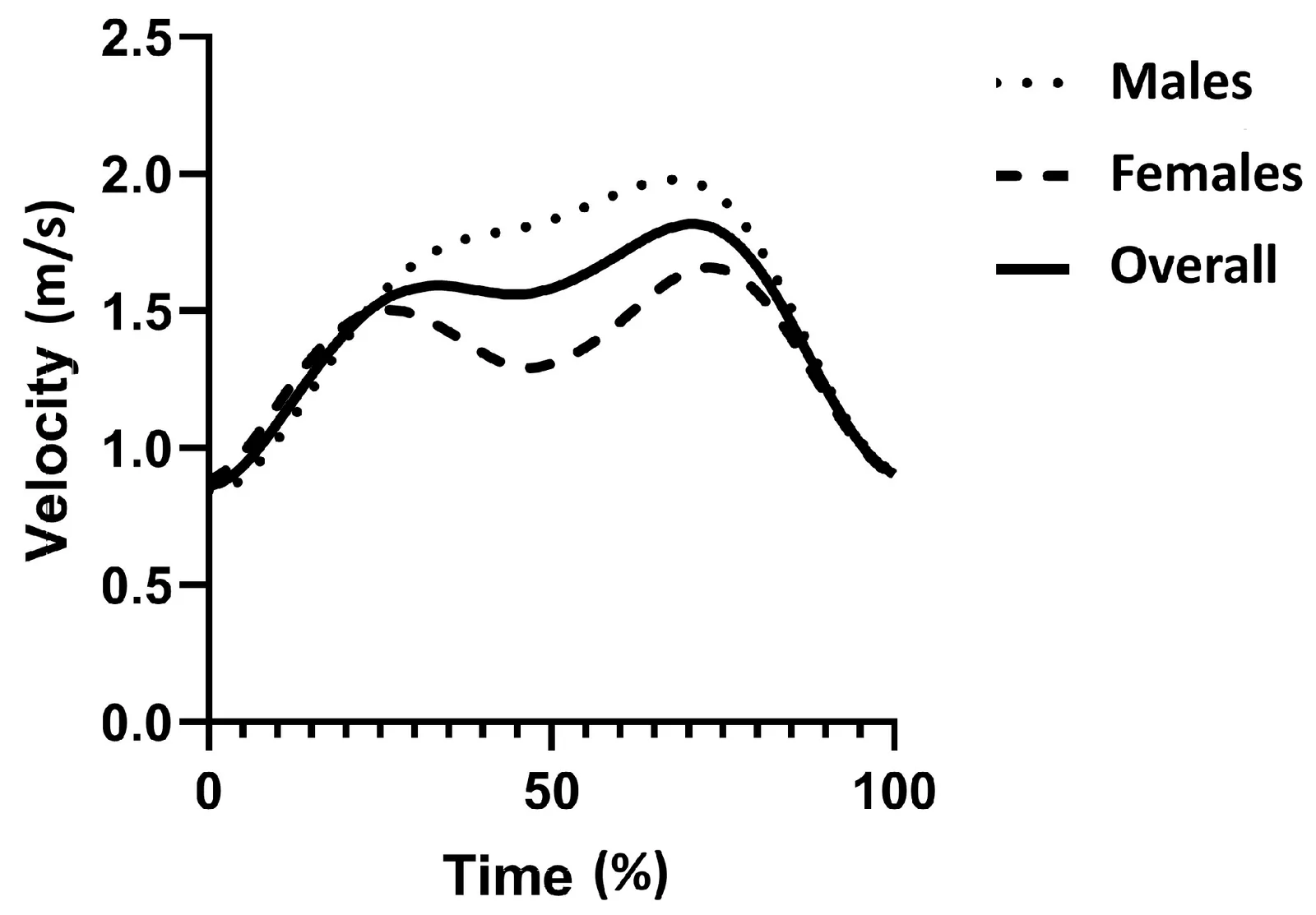
Velocity–time curve of the average of the five stroke cycles measured, per sex and for the overall sample.

Before in-water data collection, the swimmers completed a warm-up session designed to enhance sprint performance [17]. Afterward, they were randomly instructed to perform three 25 m all-out trials in the butterfly stroke with a wall push-off start. To define the stroke cycles, the upper-limb actions were used. For the swimming velocity measurement, a stroke cycle was defined as the time interval between successive hand entries. A video camera (GoPro Hero Black 7, San Mateo, CA, USA) was positioned at a fixed location in the mid-section of the swimming pool (between the 10th and 20th meters). This was synchronized with the speedometer to capture video recordings of the swimmers in the sagittal plane, identifying the swimmers’ hand entry to set the beginning and end of the stroke cycle. A 30-min rest period was provided between trials to ensure full recovery. The trial with the fastest swimming velocity was selected for analysis. Five consecutive stroke cycles were measured and analyzed between the 10th and 20th meters. This represents 105 stroke cycles in total. Between such markers, swimmers were instructed to perform stroke cycles while holding their breath to minimize disruptions in stroke coordination or technique that might influence swimming velocity [18]. The swimming velocity and the intra-cyclic variation in swimming velocity (dv) of five consecutive stroke cycles were analyzed. Figure 1 illustrates the velocity–time curve, which in this case is calculated as the average of the five stroke cycles.

### 2.3. Data Collection and Curation

Swimming velocity (in m/s) was measured with a speedometer (SpeedRT, ApLab, Rome, Italy). The device recorded displacement and velocity data at a sampling frequency of 100 Hz. The velocity–time data series were subsequently imported into signal-processing software (AcqKnowledge v. 3.9.0, Biopac Systems, Santa Barbara, CA, USA). Following residual analysis, the signal was processed using a fourth-order Butterworth low-pass filter with a cut-off frequency of 5 Hz. The dv was calculated based on two approaches: (i) as the coefficient of variation (CV = standard deviation/mean ∙ 100, in %—dv1) [1], and (ii) as the difference between the maximum and minimum instantaneous velocity (range) (in m/s—dv2) [14]. Before this analysis, each stroke cycle was temporally normalized to 0–100% of its duration using a custom R routine [19]. Linear interpolation was used to resample each cycle to 101 equally spaced points (0–100%, at 1% intervals), providing a common time base for point-by-point comparison and averaging of the velocity profiles. This procedure provided a common relative time base for cycles of different absolute durations, enabling point-by-point comparison and averaging of the velocity profiles across cycles and swimmers. Without temporal normalization, differences in stroke-cycle duration would preclude direct comparison of the corresponding velocity profiles. For each one of the 105 stroke cycles, the swimming velocity, dv1 (CV), and dv2 (range) were calculated.

### 2.4. Statistical Analysis

The mean plus one standard deviation was calculated as descriptive statistics. Data distribution was analyzed with the Kolmogorov–Smirnov test. A logarithmic transformation (LN) was used because both dv measures showed non-normal distributions. This allowed us to obtain approximately normal distributions and satisfy the assumptions of the subsequent statistical analyses. The transformation was not intended to make the two measures dimensionally equivalent, as dv1 and dv2 remained conceptually and mathematically distinct. The subsequent analysis was only done on the transformed data. The consistency and relationship between dvs were determined based on: (i) the IntraClass Correlation Coefficient (ICC) and (ii) simple linear regression, respectively. For the ICC, the two-way mixed model with the “consistency” definition was used [20]. The qualitative interpretation was performed as follows: (i) poor, if ICC < 0.5; (ii) moderate, if 0.5 ≤ ICC < 0.75; (iii) good, if 0.75 ≤ ICC < 0.90; and (iv) excellent, if ICC > 0.90 [20]. For the simple linear regression, the coefficient of determination (R^2^) was calculated. The relationship was considered: (i) very weak if R^2^ < 0.04; (ii) weak if 0.04 ≤ R^2^ < 0.16; (iii) moderate if 0.16 ≤ R^2^ < 0.49; (iv) high if 0.49 ≤ R^2^ < 0.81; and (v) very high if 0.81 ≤ R^2^ < 1.0 [21].

## 3. Results

Table 1 presents descriptive statistics for swimming velocity and its intra-cyclic variation, based on the two assessment methods (dv1 and dv2), for each sex, with both sexes plotted together. Velocity variation variables with logarithmic transformation (LN—which were used for analysis) are also presented. Males presented faster swimming velocities and greater dvs than their female counterparts (Table 1). The ICC between dvs was excellent in all three conditions (overall: ICC = 0.972, *p* < 0.001; males: ICC = 0.978, *p* < 0.001; females: ICC = 0.955, *p* < 0.001).

**Figure 2:**
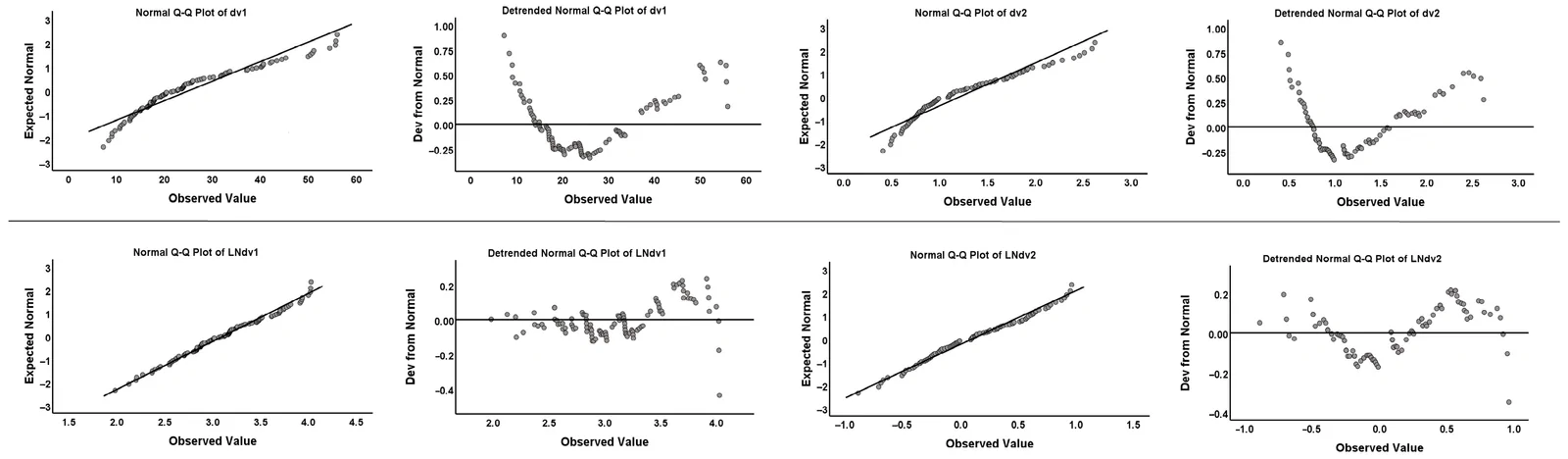
Distribution plots for non-transformed (dv1 and dv2—upper panels) and transformed data (LNdv1 and LNdv2—bottom panels).

Figure 2 presents the distribution plots for the non-transformed (dv1 and dv2) and logarithmically transformed data (LNdv1 and LNdv2). The distribution of dv1 and dv2 was further examined using normal and detrended Q-Q plots. For the untransformed variables, both indices showed systematic departures from the expected normal distribution, particularly at the tails. Following logarithmic transformation, the observations aligned more closely with the theoretical normal line, while deviations in the detrended Q-Q plots were substantially reduced. These plots, therefore, supported the use of logarithmically transformed dv1 (LNdv1) and dv2 (LNdv2) values in subsequent analyses.

**Figure 3:**
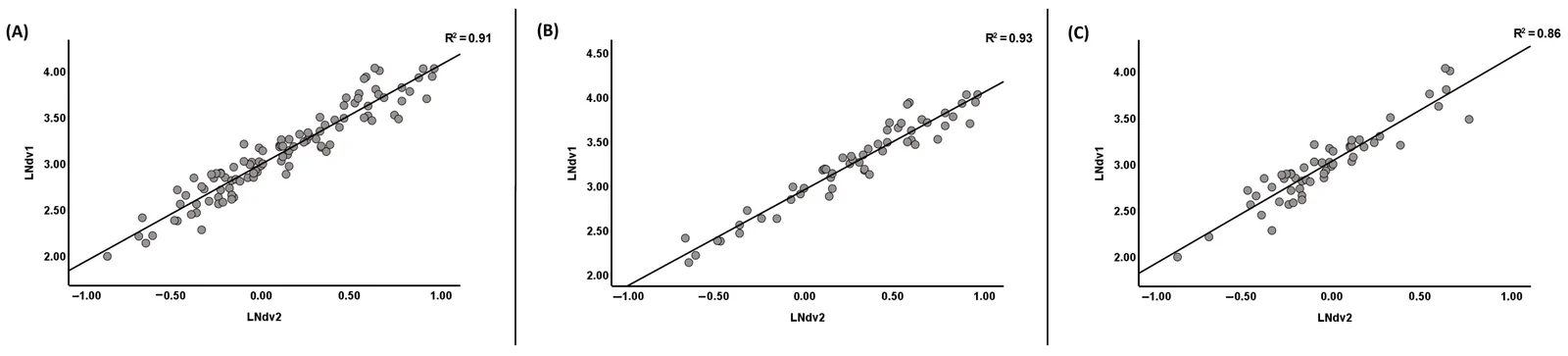
Simple linear regression between the two methods used to calculate the dv. (**A**)—overall sample; (**B**)—males; (**C**)—females.

Figure 3 presents the linear regressions between dvs, per sex, and for the overall sample. For all three conditions, the linear regression between methods was very high (overall: R^2^ = 0.93, *p* < 0.001; males: R^2^ = 0.91, *p* < 0.001; females: R^2^ = 0.86, *p* < 0.001).

## 4. Discussion

### 4.1. Main

The aim of this study was to assess the association and consistency between two methods for measuring the dv. The main findings were that the ICC between the two dv measures was excellent, and the simple linear regression was very high. Although CV (LNdv1) and range (LNdv2) are based on different mathematical formulations, the results demonstrated a very high association and excellent consistency between the two indices. The CV expresses the standard deviation of all instantaneous velocity values relative to the mean velocity, whereas the range represents only the absolute difference between the maximum and minimum instantaneous velocities. Nevertheless, both measures increase with the overall magnitude of the velocity fluctuations. Therefore, when swimmers present broadly similar velocity–time waveform shapes, a greater peak-to-trough amplitude is also likely to be accompanied by greater overall dispersion. This provides a mathematical and biomechanical explanation for the very high association and excellent consistency observed between LNdv1 and LNdv2. Thus, contrary to the initial expectation of limited consistency, both methods yielded highly concordant estimates of between-swimmer differences in dv. However, this does not indicate that the two measures are mathematically equivalent. While CV (LNdv1) reflects the relative dispersion of velocity throughout the entire stroke cycle, range (LNdv2) is determined only by the maximum and minimum instantaneous velocity values.

For the present study, two analyses were employed to assess the association and consistency between the two most commonly used methods to measure dv: CV (LNdv1) and the difference between the maximum and minimum instantaneous velocities, or range (LNdv2) [3]. Overall, the two approaches were highly consistent and strongly associated after logarithmic transformation. The excellent consistency ICC indicates that the two indices produced a very similar ranking of swimmers, meaning that swimmers with greater LNdv1 generally also presented greater LNdv2. The very high regression coefficient further demonstrates a strong linear relationship between the measures. Together, these results provide converging evidence that LNdv1 and LNdv2 yielded concordant information on between-swimmer differences in velocity fluctuations under the present experimental conditions. However, because they are expressed in different units and are derived from different components of the velocity signal, they should not be considered numerically identical or universally interchangeable.

Another aspect of dv was the considerable dispersion of values observed with both approaches (i.e., LNdv1 and LNdv2). Previous studies have also reported substantial differences in dv values, which may reflect differences in swimming performance and technical characteristics among swimmers [3,22]. In the present study, five stroke cycles were analyzed separately for each swimmer; therefore, the overall dispersion of dv values may reflect both differences among swimmers and cycle-to-cycle differences within the same swimmer. These sources of variation were not separately quantified. Both measurements showed non-normal distributions, as indicated by visual inspection of histograms and Q-Q plots and by the Kolmogorov–Smirnov test (*p* < 0.05). Therefore, the data were logarithmically transformed before subsequent analyses. However, the present analyses do not allow determination of the specific biomechanical factors underlying the observed dispersion or distributional shape. Within a stroke cycle, velocity varies with the alternating contributions of propulsive and resistive forces, resulting in distinct acceleration and deceleration phases [3,4]. Thus, the velocity–time profile need not show a symmetrical pattern around the mean velocity. Nevertheless, the present study did not examine whether these within-cycle characteristics explain the positive skewness observed in the dv values.

Once again, this may be due to an inherent, individual motor strategy [8]. Moreover, it was recently claimed that all dv measurements fail to consider the role of drag, and thus, limit their competence to assess mechanical swimming efficiency [23]. This can be another reason for the high variability of the dv, indicating that the variable is very intrinsically/individually driven. Indeed, it has been claimed that variability in human movement (often dismissed as “noise”) may carry meaningful information about motor control and skill adaptation [24]. Rather than viewing variability as an undesirable source of error, Preatoni and co-workers argued that it reflects the system’s ability to flexibly adjust to internal and external constraints, thereby contributing to stability and performance efficiency [24]. In sports contexts, this means that fluctuations in movement patterns are not simply random disturbances but can reveal how athletes explore different coordination strategies to optimize performance and reduce injury risk. Therefore, noise should not be neglected in performance analysis; rather, it should be viewed as an integral component of skilled movement behavior, offering insights into the athlete’s adaptability, consistency, and overall motor learning [25,26].

This assumption can be linked to the dv’s outliers that may be neglected or removed. In this case, a non-normal distribution was observed, necessitating a logarithmic transformation. What might appear as an outlier could represent a functional adaptation or a response to subtle perturbations within the movement system [27]. Therefore, in the dv analysis, instead of treating all deviations as errors, researchers should carefully evaluate whether data points considered outliers reflect noise or meaningful aspects of coordination variability. Outlier data points can be evaluated using a combination of statistical and biomechanical criteria, including consistency across repeated trials, temporal localization within specific stroke phases, and sensitivity analyses with and without their inclusion [28]. Deviations that are structured, phase-dependent, and reproducible are more likely to represent meaningful coordination variability rather than random measurement noise. This will ensure that valuable information about the dynamics of skilled performance is not lost. In this case, the range can be strongly influenced by isolated outliers or random spikes that arise for unexplained reasons and do not reflect the overall behavior of the velocity–time curve [29]. Overall, CV and range describe variability differently and reflect distinct mathematical properties of the same signal. Their consistency and association can be explained by the fact that both are sensitive to the general magnitude of velocity fluctuations. For broadly similar velocity–time profiles, an increase in the difference between the maximum and minimum velocities is generally accompanied by an increase in the standard deviation. However, the proportional relationship between these measures is not fixed, as it depends on the mean velocity, waveform shape, the distribution of instantaneous velocity values, and the presence of isolated peaks or troughs. The range is determined solely by the extreme values, whereas the CV expresses variability relative to the mean and is influenced by the overall dispersion. For that reason, researchers should be aware that a very high association and excellent consistency do not necessarily imply redundancy, because the two indices may provide complementary interpretations of the same velocity–time signal.

### 4.2. Overview

In summary, fluctuations in swimming during the stroke cycle have been extensively studied and remain of paramount interest to researchers and coaches. Notwithstanding, this topic still needs deeper interpretation to fully understand its intra- and inter-subject variability. Despite a strong association and excellent consistency between measurements, it must be mentioned that the dv measured as being the CV (LNdv1) may present a deeper interpretation because: (i) it represents the overall underlying variability pattern, instead of just two discrete points; (ii) is less sensitive to noise (considering the entire time-series and not discrete datapoints can smooth out hypothetically random events or data handling procedures); (iii) statistically robust, i.e., standard deviation captures total dispersion, and; (iv) is good for between-subjects and within-subjects comparisons because it account for all fluctuations (acceleration and deceleration phases). On the other hand, using both methods may provide complementary information about dv. In other words, a close association and consistency do not necessarily imply mathematical equivalence or redundancy. Two measures can covary strongly because they respond to the same underlying fluctuation magnitude while still describing different properties of the signal. In the butterfly stroke, the CV (LNdv1) and range (LNdv2) yielded similar interpretations of swimming velocity fluctuations. The butterfly stroke is ideal for analyzing swimmers’ dv because of its distinct propulsion and recovery phases, and pronounced fluctuations make changes in velocity more detectable and meaningful. However, this association and consistency may not hold across all swim strokes. For instance, front crawl is characterized by more complex velocity fluctuations, with multiple fluctuations occurring within each stroke due to alternating upper- and lower-limb actions and their coordination. These additional fluctuations may increase the likelihood of isolated peaks or transient velocity changes, which can disproportionately influence the range while having a different impact on the CV.

### 4.3. Limitations and Future Studies

As main limitations, the relatively small sample size limited sex-stratified analyses and generalizability (nonetheless, 105 stroke cycles were analyzed). Only butterfly swimmers were included, and only the fastest trial was analyzed under a non-breathing condition, which may limit ecological validity. In addition, five repeated stroke cycles from the same trial were used and were therefore not fully independent. Finally, both dv assessment methods were derived from the same velocity signal and should be interpreted as different mathematical descriptions of the same underlying data rather than independent measurement methods. Therefore, future studies should focus on verifying the association and consistency between these two methods of measuring velocity fluctuations across different swim strokes and race paces. They should also include larger and more diverse samples, examine breathing patterns, consider multiple trials, and assess whether similar relationships between dv methods are observed across independent datasets and measurement conditions.

## 5. Conclusions

The two most commonly used methods for calculating dv (CV and the difference between the maximum and minimum instantaneous velocities—range) yielded an excellent ICC for consistency and a very high linear association. Therefore, despite being based on different mathematical formulations, LNdv1 and LNdv2 provided highly concordant information regarding differences in intra-cyclic velocity variation in butterfly swimming. However, this consistency and association should not be interpreted as mathematical equivalence or universal interchangeability. The CV describes relative dispersion across the entire velocity–time series, whereas the range describes the absolute peak-to-trough amplitude. Thus, the two measures may yield similar overall interpretations while still representing distinct and potentially complementary characteristics of the velocity signal.

## Figures and Tables

**Table 1:** Descriptive statistics (mean ± standard deviation—SD) of swimming velocity and intra-cyclic variation in swimming velocity based on the two different methods of assessment, per sex and both sexes plotted together.

	Mean ± SD		
	Males	Females	Overall
**Velocity (m/s)**	1.50 ± 0.16	1.38 ± 0.15	1.45 ± 0.17
**dv1 (%)**	28.9 ± 12.8	20.8 ± 10.1	25.1 ± 12.3
**dv2 (m/s)**	1.4 ± 0.6	1.0 ± 0.4	1.2 ± 0.5
**LNdv1 (a.u.)**	3.3 ± 0.5	2.9 ± 0.4	3.1 ± 0.5
**LNdv2 (a.u.)**	0.3 ± 0.4	-0.1 ± 0.3	0.1 ± 0.4

dv1—velocity fluctuation as the coefficient of variation; dv2—velocity fluctuation as the difference between the maximum and minimum instantaneous velocity (range); LNdv1—logarithmic transformation of velocity fluctuation as the coefficient of variation; LNdv2—logarithmic transformation of velocity fluctuation as being the difference between the maximum and minimum instantaneous velocity (range).

## Data Availability

The data presented in this study are available on request from the corresponding author. The data are not publicly available due to privacy or ethical restrictions.

## Abbreviations

The following abbreviations are used in this manuscript:

IVV	Intra-cyclic variation in swimming velocity
dv	Speed fluctuation
CV	Coefficient of variation
dv1	Speed fluctuation as the coefficient of variation
dv2	Speed fluctuation as the difference between the maximum and minimum instantaneous velocity
LN	Logarithmic transformation
ICC	IntraClass Correlation Coefficient
95CI	95% confidence intervals
